# Fetal nuchal cystic hygroma associated with aortic coarctation and trisomy 21: a case report

**DOI:** 10.4076/1757-1626-2-8280

**Published:** 2009-08-04

**Authors:** Sohei Kitazawa, Kiyoshi Mori, Takeshi Kondo, Riko Kitazawa

**Affiliations:** Division of Pathology (Diagnostic Molecular Pathology Unit), Kobe University Graduate School of Medicine 7-5-1 Kusunoki-cho, Chuo-ku, Kobe 650-0017Japan

## Abstract

We report a case of fetal nuchal cystic hygroma associated with aortic coarctation and trisomy 21. A stillborn baby, delivered at 15 weeks and 5 days of gestation, had a huge nuchal cystic hygroma. Autopsy revealed aortic coarctation of the periductal type with patent ductus arteriosus, endocardial cushion defect and left ventricular hypoplasia. Trisomy 21 was evident by karyotyping. Macroscopically, while an apparent association of nuchal cystic hygroma and aortic coarctation resembled Turner syndrome, histopathological findings were those typically seen in trisomy 21: numerous dilated lymphatics in the subcutaneous tissue with severe mesenchymal edema, and an enlarged jugular lymphatic sac.

## Introduction

Various lymphatic abnormalities cause jugular lymphatic distension resulting in nuchal edema and cystic hygroma [1,2]. Increased nuchal translucency by ultrasound examination directly reflects the presence of nuchal edema and cystic hygroma, and is regarded as a marker for aneuploidy such as Turner syndrome or trisomy of various chromosomes [3,4]. Here, the rare occurrence of a huge congenital fetal nuchal hygroma associated with complex cardiac anomalies including aortic coarctation in a trisomy 21 case is described.

## Case presentation

**Figure 1.**
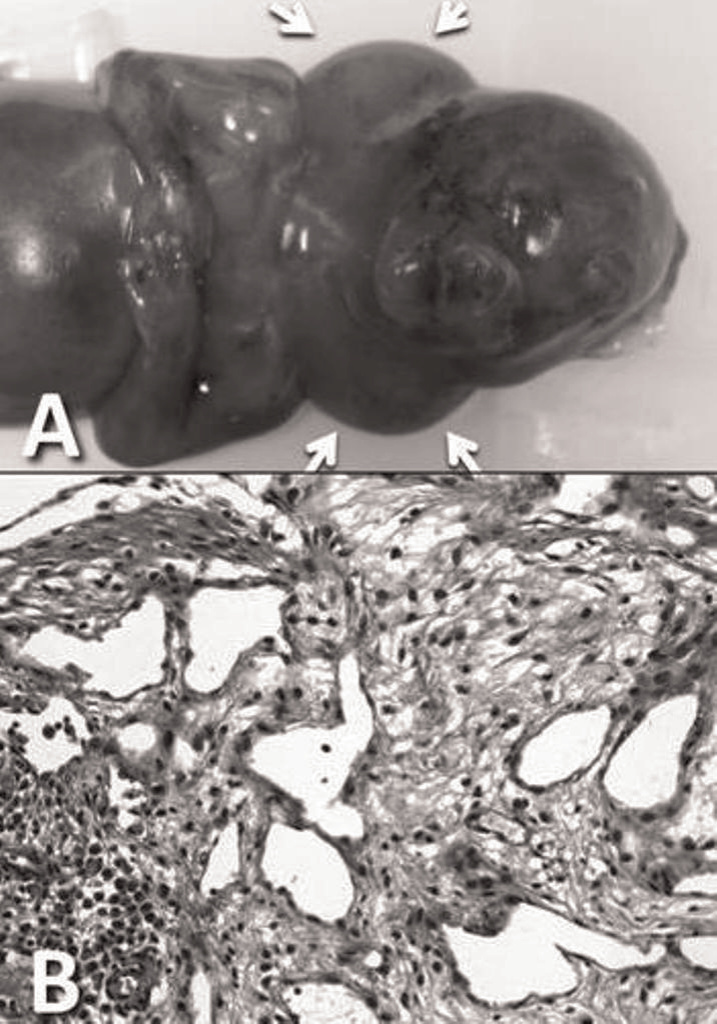
Macroscopic and microscopic findings of the cystic hygroma. **(A)** a huge nuchal cystic mass measuring 20 × 15 mm is observed around the neck (arrows). Externally, no other apparent malformations are noted. **(B)** Histopathological examination revealed numerous dilated lymphatics with proliferation of spindle-shaped mesenchymal cells and edema in the subcutaneous area (HE, ×200).

**Figure 2.**
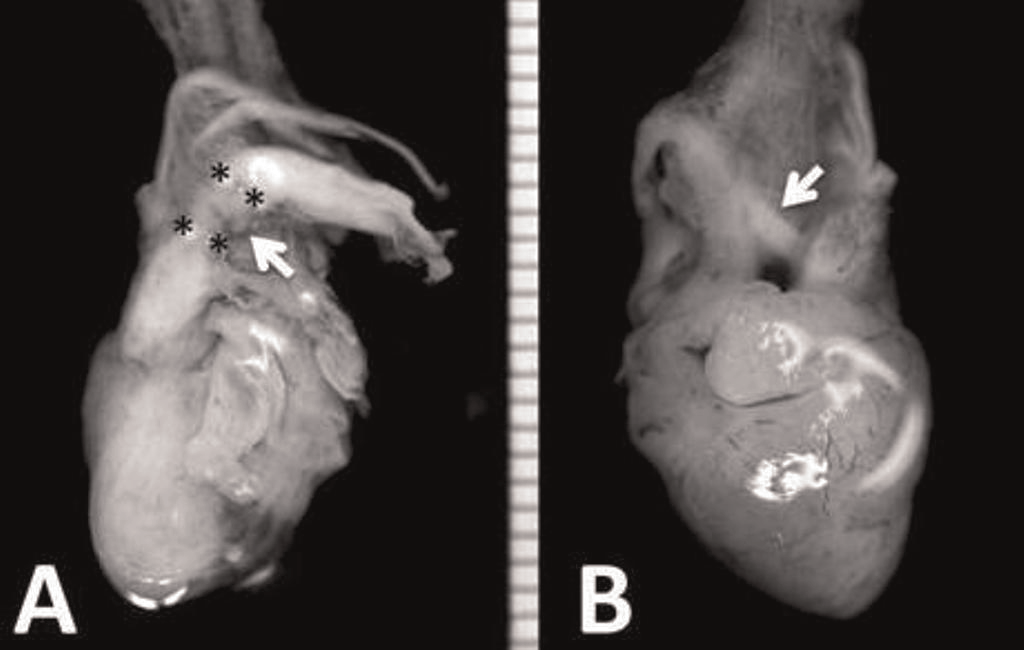
The heart, from anterior **(A)** and posterior **(B)** views. The heart weighted 1.5 g and showed coarctation of the aortic arch **(A, asterisks)** with prominent patent ductus arteriosus **(A and B, arrows)**.

A stillborn Japanese baby, delivered at 15 weeks and 5 days of gestation, weighed 145 g and measured 16.6 cm long. At autopsy, no apparent malformation was noted except for a huge nuchal cystic mass measuring 20 × 15 mm, (Figure 1A, arrows) from the external aspect. Histopathological examination of the nuchal mass revealed numerous dilated lymphatics with proliferation of spindle-shaped mesenchymal cells and edema in the subcutaneous area (Figure 1B, HE, ×200). The heart, weighing 1.5 g, showed coarctation of the aortic arch (Figure 2A, asterisks) with prominent patent ductus arteriosus (Figure 2A and 2B, arrows). Endocardial cushion defect and slight left ventricular hypoplasia were also noted. Karyotypic analysis of the fetal blood showed trisomy of chromosome 21 (data not shown), and a definitive diagnosis of nuchal cystic hygroma associated with trisomy 21 and aortic coarctation was made.

## Discussion

Nuchal edema or cystic hygroma observed as increased nuchal translucency by ultrasound examination is regarded as a marker for aneuploidy. Since nuchal cystic hygroma is frequently associated with Turner syndrome with aortic coarctation, nuchal translucency is used as a marker for the antenatal diagnosis of aortic coarctation also [5]. Indeed, studies by Ph Descamps et al. have shown that Turner syndrome (45X) and Down’s syndrome (trisomy 21) comprise nearly half the cases of nuchal cystic hygroma [3]. The mechanism whereby each chromosomal abnormality develops nuchal edema or cystic hygroma is though to be different, however [6]. Histopathologically, cases with Down’s syndrome are also different from those with Turner syndrome in that the former have an enlarged jugular lymphatic sac with numerous dilated lymphatics in the subcutaneous tissue with severe mesenchymal edema [6]. In our present case, while an apparent association of nuchal cystic hygroma and aortic coarctation suggested the presence of Turner syndrome, histopathological findings were those typically seen in trisomy 21.
